# Reduction of intraarticular adhesion of knee by local application of rapamycin in rabbits via inhibition of fibroblast proliferation and collagen synthesis

**DOI:** 10.1186/s13018-016-0375-0

**Published:** 2016-04-19

**Authors:** Shuai Zhao, Yu Sun, Xiaolei Li, Jingcheng Wang, Lianqi Yan, Hui Chen, Daxin Wang, Jihang Dai, Jun He

**Affiliations:** Department of Orthopedics, Xiangya Second Hospital, Central South University, Changsha, Hunan 410012 China; Department of Orthopedics, Clinical Medical College of Yangzhou University, Nantong West Road 98, Yangzhou, Jiangsu 225001 China; Orthopedics Institute, Subei People’s Hospital of Jiangsu Province, Yangzhou, Jiangsu 225001 China

**Keywords:** Rapamycin, Intraarticular adhesion, Fibroblast, Collagen synthesis

## Abstract

**Background:**

The formation of intraarticular adhesion is a common complication after total knee arthroplasty or anterior cruciate ligament reconstruction. Previously, little research was reported regarding whether the local application of rapamycin (RAPA) could reduce intraarticular adhesion following knee surgery. In our present study, we determined the therapeutic effect of RAPA by local application on the reduction of intraarticular adhesion following knee surgery in rabbits.

**Methods:**

In this study, we built the model of knee surgery according to a previous study. The decorticated areas of the cortical bone were exposed and covered with cotton pads soaked with different concentrations of RAPA or physiological saline for 10 min. All of the rabbits were euthanized 4 weeks after the surgery. Macroscopic evaluation of the hydroxyproline content, the histological morphological analysis and collagen density and fibroblast density were used to evaluate the effect of RAPA on reducing intraarticular adhesion.

**Results:**

The results shown that RAPA could significantly inhibit the proliferation of fibroblasts and reduce collagen synthesis; in the rabbit model of knee surgery, there were weak scar tissues around the decorticated areas in the 0.2 mg/ml RAPA group; moderate scar tissues were found in the 0.1 mg/ml RAPA group. However, severe fibrous adhesions were found in the 0.05 mg/ml RAPA group and the control group. The hydroxyproline content and the fibroblast density in the 0.2 mg/ml and 0.1 mg/ml RAPA groups were significantly less than those of the control group.

**Conclusions:**

We concluded that the local application of RAPA could reduce intraarticular adhesion after knee surgery in the rabbit model; this effect was mediated by inhibition of fibroblast proliferation and collagen synthesis, which may provide a new method for reducing intraarticular adhesion after clinical knee surgery.

## Background

Intraarticular adhesion after knee trauma or ligament surgery is a disabling complication in orthopaedics [1, 2]. Restrictive adhesions after knee surgery cause many issues for patients who often suffer joint stiffness and pain. Because intraarticular adhesion will further affect the biomechanics of the knee, it will accelerate cartilage degeneration and eventually lead to irreversible damage [3–5].

Currently, the underlying mechanisms of intraarticular adhesion remain unclear. However, some researchers think that many cytokines and growth factors can contribute to fibrous tissue hyperplasia, which may be partially responsible for the formation of intraarticular adhesion [6–8]. It is generally recognized that trauma itself and surgical trauma will activate inflammatory responses. Subsequently, cytokines and growth factors are produced, and they will stimulate fibroblasts to proliferate excessively. Fibroblasts produce collagen and release extracellular matrix, which ultimately results in the formation of intraarticular adhesion [9–11].

Several approaches, such as minimally invasive surgery and careful haemostasis, have been used to prevent intraarticular adhesion [12, 13]. Moreover, researchers have reported a variety of medicines and artificial synthetic materials that have been used to prevent intraarticular adhesions [14–16]; however, limited or variable success was achieved. Therefore, new solutions to solve this problem are still greatly needed.

Rapamycin (RAPA) is a new cyclic macrolide antibiotic that is widely used to inhibit organ allograft rejection (such as corneal allograft and renal transplantation) because of its lack of renal toxicity in clinical trials [17, 18]. The local application of RAPA has been proven to be effective in inhibiting neointimal hyperplasia and vein graft restenosis in experimental vein grafts [19, 20]. Many studies have reported that RAPA has proven to be effective in preventing various reasons caused pulmonary fibrosis, liver fibrosis and peritoneal fibrosis [21–23]. Recently, RAPA has shown the effect when used systemically to prevent scleroderma and corneal scarring after photorefractive keratectomy [24–26]. However, whether it has the effect of preventing intraarticular adhesion after knee surgery is unknown.

In the present study, we intended to illustrate the effect of RAPA on the reduction of intraarticular adhesion after knee surgery in the rabbit model. If the local application of RAPA proves to be an effective solution, it will provide a novel idea for reducing intraarticular adhesion after knee surgery.

## Methods

### Animals

Forty-eight mature male New Zeal rabbits weighing 3.5 to 4.0 kg were purchased from Shanghai Laboratory Animal Center (Shanghai, China). All of the animals were housed in a controlled environmental condition to acclimate to the environment for 1 week before surgery. The animals were given normal chow and water. The rabbits were randomly divided into four groups as follows (12 rabbits in each group): A group: 0.2 mg/ml RAPA group; B group: 0.1 mg/ml RAPA group; C group: 0.05 mg/ml RAPA group; and D group: control (saline) group.

### Reagents

RAPA was obtained from Santa Cruz Biotechnology (Santa Cruz, CA).

### Animal model

The animal models of intraarticular adhesions were performed according to the procedures of previous research [1, 27]. Briefly, after animals were anaesthetized by intravenous administration of 10 % pentobarbital sodium (4 ml/kg), the hairs around the left knee joint were shaved and the exposed skin was sterilized with iodophor three times. A medial parapatellar approach was used to open the medial and lateral sides of the femoral condyle. Then, approximately 1 × 1 cm^2^ of the bone cortex was removed from both sides of the femoral condyle, and the underneath cancellous bone was exposed. The articular cartilage was left intact.

### Local application of drugs

Cotton pads soaked with various concentrations of 0.2, 0.1, and 0.05 mg/ml RAPA or normal saline were applied for 10 min to the decorticated areas of the femoral condyle (15 × 15 mm) after satisfactory haemostasis. The surrounding tissues were protected using wet gauze to avoid exposure with the agent. After the cotton pads were removed, the decorticated areas of the femoral condyle were washed immediately with enough saline to remove the remaining RAPA. The articular capsule and skin were closed with silk sutures, and the surgical knee joints were fixed extraarticularly in the fully flexed position with a Kirschner wire for 4 weeks. Cefazolin sodium (50 mg/kg) was administered intramuscularly to prevent infection postoperatively for 3 days. The animals were bred individually with free access to standard chow and water after surgeries.

### Macroscopic evaluation

The macroscopic evaluation was performed postoperatively after 4 weeks. Four rabbits were randomly selected from each group, and anaesthetized by intravenous administration of 10 % pentobarbital sodium (4 ml/kg). The intraarticular adhesions were evaluated by three professional pathologists who were blinded to the treatment groups according to the following visual scoring system [28]: grade 0: no adhesions; grade 1: weak, mild, filmy adhesions that can be easily dissected by minimal manual traction; grade 2: moderate adhesions that can be dissected by manual traction; and grade 3: dense and firmly fibrous adhesions that must be surgically removed.

### Biochemical analysis of the hydroxyproline content

Four rabbits were euthanized with an overdose of urethane and served for further biochemical analysis. The hydroxyproline content in the adhesion tissue was determined according to the method of Woessner [29]. The knee was opened, and approximately 20 mg wet weight of the adhesion tissue was obtained from the centre of the decorticated areas. The samples were lyophilized, ground separately and hydrolysed. Then, the samples were neutralized with 2.5 N NaOH on the indication of methyl red. The chloramine T was added to the hydrolysed samples and hydroxyproline standards of four known concentrations. After incubation at room temperature, the hydroxyproline developer was added to the samples and the standards. The absorbance of the solution was determined at 558 nm with the spectrophotometer, and the levels of hydroxyproline per milligram of scar tissue were calculated according to the standard curve that constructed by the serial concentration of commercial hydroxyproline.

### Histological analysis

The histological analysis was performed in four groups postoperatively after 4 weeks. Four rabbits were selected from each group and euthanized with an overdose of urethane. The knee joints were excised including all of the connective tissues and the fibrotic adhesive scar. The samples were fixed in 10 % buffered formalin for 1 week and decalcified for 2 weeks. The tissues were embedded in paraffin, and transverse sections perpendicular to the femoral axis were stained with haematoxylin–eosin (HE). The intraarticular scar adhesions were evaluated under the light microscope with a magnification of ×200. Histological images (magnification ×200) of the sections stained with HE from each rabbit were obtained. Three counting areas in the scar tissue close to the bottom of the decorticated areas were selected, and each was approximately 100 × 100 μm. The density of the fibroblasts was calculated, and the density of the fibroblasts for each section was defined as the mean number from three fields.

### Densitometric analysis of collagen tissue

The optical density of collagen was observed using a light microscope at a magnification of ×200. A densitometric analysis of the collagen tissue was also performed. The sections stained with Masson’s trichrome were photographed using a light microscope (Olympus BX50, Japan) connected to a CCD camera (Olympus DP70, Japan). The optical density value of the positively stained collagen was determined using Image Pro Plus 6.0 image analysis software.

### Statistical analysis

The statistical analysis was performed using SPSS software (version 13.0). The data were expressed as mean ± standard deviation values. The differences were considered statistically significant when *P* < 0.05.

## Results

### Macroscopic evaluation of intraarticular adhesion

The surgery was well tolerated by all animals, and there was no case of wound infection or disturbance of wound healing in any of the rabbits. The macroscopic observation showed that soft or weak fibrous adhesion was observed around the decorticated areas of the femoral condyle in 0.2 mg/ml RAPA group. In the 0.1 mg/ml RAPA group, the decorticated areas were covered with moderate scar adhesion, which could be dissected by manual traction. However, dense and tenacious fibrous adhesions were observed around the decorticated areas of the femoral condyle in the 0.05 mg/ml RAPA and control group, which were difficult to dissect because the scar adhesions were accompanied with bleeding. The degree of intraarticular adhesions was evaluated according to the visual scoring system, and the results are shown in Table 1.

**Table 1:** Knee intraarticular adhesion grade was based on the visual scoring system

Group	Grade			
	0	1	2	3
Saline	0	0	0	4
0.05 mg/ml	0	0	1	3
0.1 mg/ml	2	2	0	0
0.2 mg/ml	3	1	0	0

Four rabbits were selected from each group. The values within the table represent the number of rabbits

### Biochemical analysis of hydroxyproline content

**Fig. 1:**
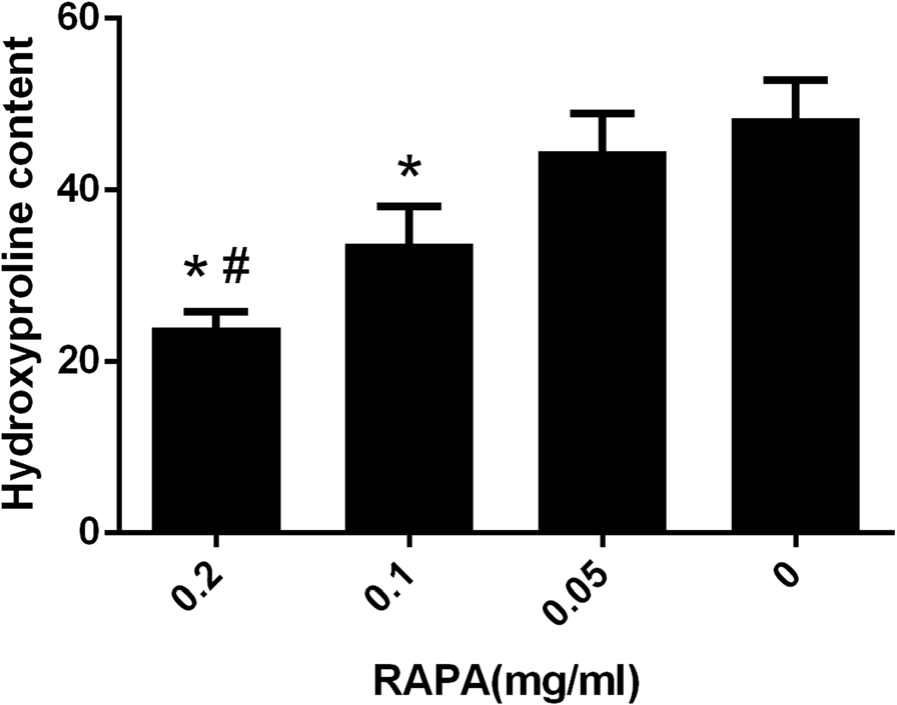
Hydroxyproline content in intraarticular scar tissue in the RAPA-treated groups and the control group. The hydroxyproline content is expressed as micrograms per milligram (μg/mg). *P < 0.05, compared with control group; #*P* < 0.05, and the 0.2 mg/ml RAPA group compared with the other RAPA groups

The statistical analysis of the hydroxyproline content in the intraarticular scar tissue for each group is shown in Fig. 1. The hydroxyproline content in the 0.2 mg/ml RAPA group was 23.94 ± 1.84 μg/mg, which was significantly less than those in the 0.1 mg/ml RAPA group (33.75 ± 4.31 μg/mg, *P* = 0.022), the 0.05 mg/ml RAPA group (44.50 ± 4.35 μg/mg, *P* = 0.002) and the control group (48.37 ± 4.43 μg/mg, *P* = 0.001). The content in the 0.1 mg/ml RAPA group was also less than those in the 0.05 mg/ml RAPA group (*P* = 0.039) and the control group (*P* = 0.015). However, the content in the 0.05 mg/ml RAPA group showed no significant difference compared with those of the control group (*P* = 0.341).

### Histological analysis of RAPA on intraarticular adhesion

**Fig. 2:**
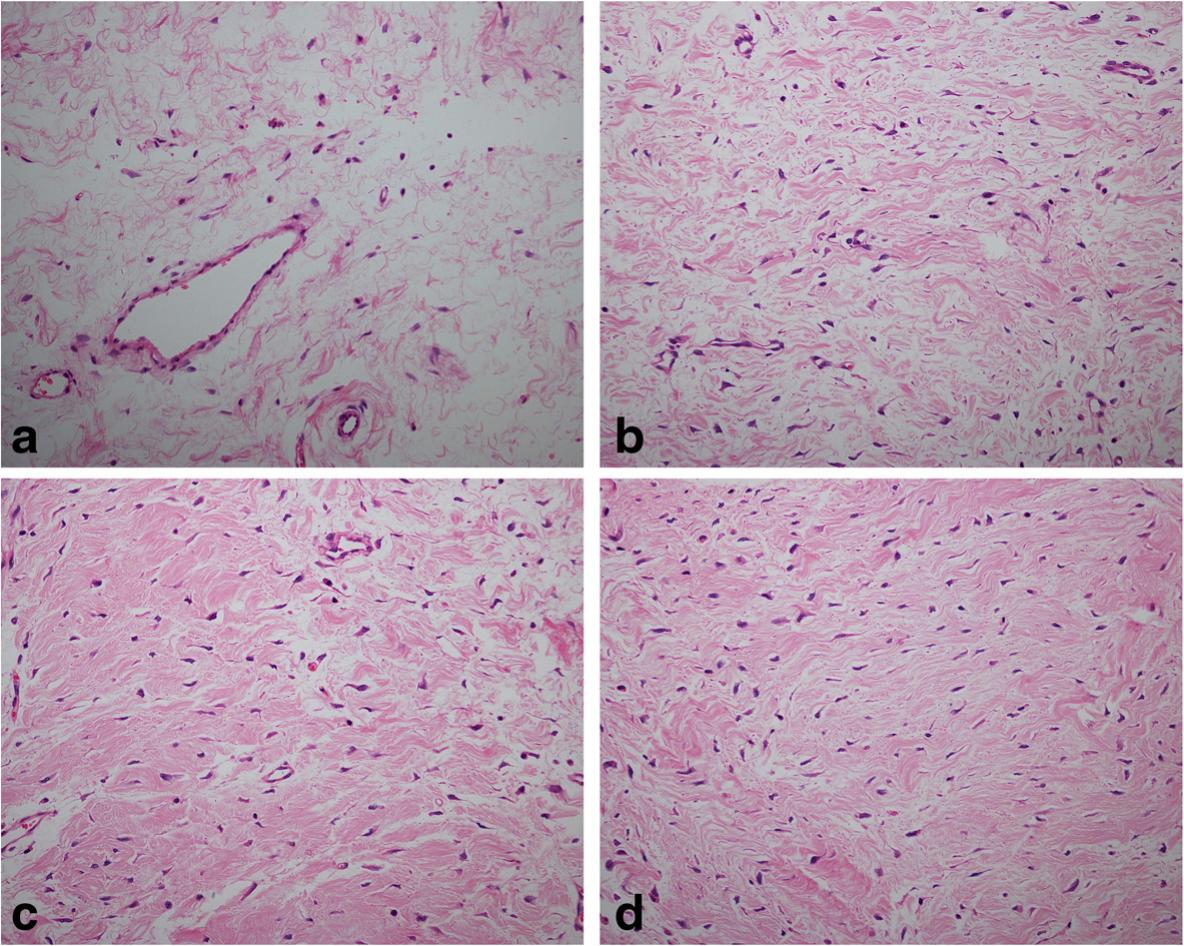
The histological view of the intraarticular adhesion issues in the decorticated areas treated with RAPA (0.2 mg/ml (**a**), 0.1 mg/ml (**b**) and 0.05 mg/ml (**c**)) and saline (**d**). Note that loose scar tissues were found in the decorticated areas treated with the 0.1 mg/ml group and 0.2 mg/ml RAPA group. Dense scar tissue was found in the decorticated areas treated with saline. The sections were stained with HE (×200)

In the control group, dense scar adhesions were found around the decorticated areas of the femoral condyle, which tethered the surrounding soft tissues to the femur. In the 0.1 mg/ml RAPA group, mild scar tissues were observed around the decorticated areas compared with those of 0.05 mg/ml RAPA group and the control group. However, loose fibrous adhesion tissue was observed in the 2.0 mg/ml RAPA group. The representative images of the HE staining of the scar tissues in each group are shown in Fig. 2.

### Collagen density analysis of RAPA on intraarticular scar adhesion

**Fig. 3:**
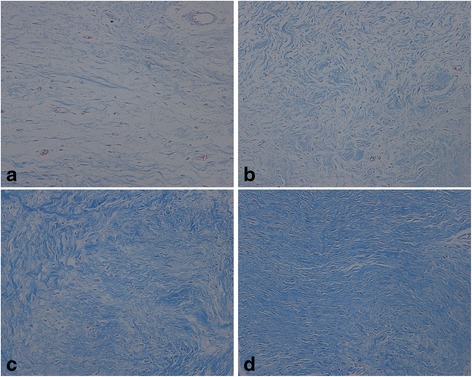
The collagen density of intraarticular adhesion tissue in the RAPA groups (0.2mg/ml (**a**), 0.1mg/ml (**b**), 0.05mg/ml (**c**) and the control group (**d**). The collagen tissues show blue in the section with Masson’s trichrome staining under the light microscope (×200). RAPA could reduce collagen synthesis and fibrosis. The density of collagen tissue in the 0.2 mg/ml RAPA group and the 0.1 mg/ml RAPA group revealed a significant decrease compared with those in the 0.05 mg/ml RAPA group and control group

**Fig. 4:**
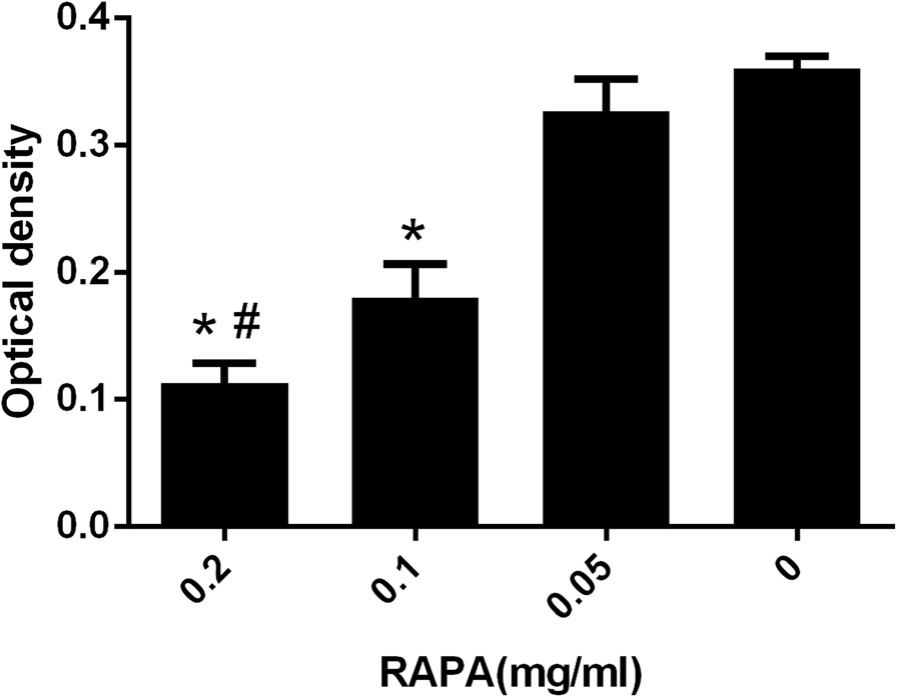
The collagen optical density in each group. *P < 0.05, compared with the control group; #*P* < 0.05, and the 0.2 mg/ml RAPA group compared with the other RAPA groups

Masson’s trichrome staining revealed that the collagen density of the intraarticular adhesion tissue in the RAPA groups coincided with HE staining. The collagen density of intraarticular tissue in the 0.05 mg/ml RAPA group (Fig. 3c) and the control group (Fig. 3d) was strong. However, the collagen density was weak in the 0.2 mg/ml RAPA (Fig. 3a) and 0.1 mg/ml RAPA group (Fig. 3b), which revealed a significant decrease compared with those in 0.05 mg/ml RAPA group and control group. Moreover, the collagen density in the 0.2 mg/ml RAPA group also revealed a decrease compared with that in the other RAPA groups. The representative images of Masson’s trichrome staining in each group are shown in Fig. 3. The statistical analysis of the optical density of the collagen tissues in each group is shown in Fig. 4.

### Effect of RAPA on the density of fibroblasts

**Fig. 5:**
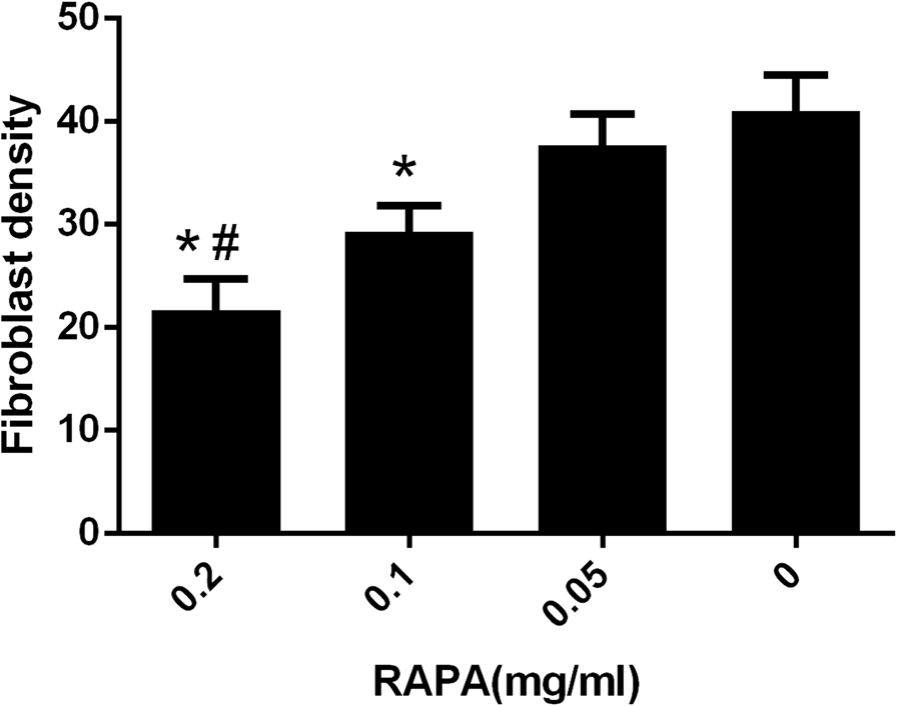
Fibroblast counts of scar adhesion tissues from each group. *P < 0.05, compared with the control group; #*P* < 0.05, the 0.2 mg/ml RAPA group compared with the other RAPA-treated groups

The fibroblast density in the scar tissue in the 0.2 mg/ml RAPA group was 21.66 ± 3.05, which was significantly less than those of the 0.1 mg/ml RAPA group (29.33 ± 2.51, *P* = 0.028), the 0.05 mg/ml RAPA group (37.66 ± 3.05, *P* = 0.003) and the control group (41.0 ± 3.46, *P* = 0.002). The density of the fibroblasts in the 0.1 mg/ml RAPA group was also less than those of the 0.05 mg/ml RAPA group (*P* = 0.022) and the control group (*P* = 0.009). However, the fibroblast density in the 0.05 mg/ml RAPA group showed no significant difference compared with those of the control group (*P* = 0.279). The statistical analysis results of the fibroblast density in the intraarticular scar tissue of each treatment group are shown in Fig. 5.

## Discussion

The study showed that the local application of 0.2 mg/ml RAPA could reduce intraarticular fibrous adhesion through inhibition of fibroblast proliferation and collagen synthesis in rabbit models. Currently, the mechanisms of intraarticular adhesion formation still remain unclear, but fibroblasts were generally recognized as a major reason for the formation of intraarticular adhesion [30, 31]. Following knee surgery, many growth factors and inflammatory cytokines will migrate to the surgical sites, where they will stimulate fibroblasts; then, fibroblasts produce the collagen fibres to repair the damaged tissues. With further changes of pathology, fibroblasts are transformed into fibrocytes, and the fibrous connective tissues finally transformed into scar tissues [27, 32]. Moderate scar tissues were helpful in repairing the damaged tissues; however, if too many scar tissues were formed, they adhere to the surrounding tissues of the joint, which results in limited motion of knee, pain, stiffness and a severe decrease in a patient’s quality of life.

Although some techniques, such as manipulation under anaesthesia, arthroscopic lysis and open debridement, are used to relieve intraarticular scar adhesion, the surgical release of scar tissues can stimulate fibrous tissue reproduction, finally resulting in unsatisfactory results of the surgeries [33, 34]. Therefore, the ideal solution seems to be the application of an anti-adhesion medicine to prevent formation of scar tissue. There are reports describing continuous decorin administration and intraarticular chitosan injection to prevent fibrous adhesion achieved some success [32, 35].

For a long time, RAPA has been known to have therapeutic effects on various types of fibrosis such as renal fibrosis, cystic fibrosis, hepatic fibrosis and peritoneal fibrosis [36–39]. Recently, it was found that RAPA could impair the process of collagen production of fibroblasts from hypertrophic and keloid scars [40, 41]; RAPA could inhibit the production of myofibroblasts and reduce corneal scarring after photorefractive keratectomy [26]. Studies demonstrated that the mammalian target of rapamycin (mTOR) plays a key role in the regulation of excessive deposition of extracellular matrix components such as collagen, fibronectin and TGF-β [40, 42]. Phosphorylation of the ribosomal protein S6, a marker of mTOR (a mammalian target of rapamycin) pathway activation, is strongly increased in hypertrophic scars and keloids; RAPA is the inhibitor of mTOR, which could downregulate the expression of collagen and fibronectin [41]. Similarly, it was reported that RAPA could modulate and downregulate the expression of collagen and MMP-1 in fibroblasts [43]. Therefore, RAPA may be a potential therapeutic agent for the treatment of intraarticular scar adhesion.

In this study, we applied different concentrations of RAPA to reduce intraarticular adhesion. Collagen is an important component of scar tissue that is primarily synthesized and secreted by fibroblasts. Moreover, hydroxyproline accounts for 12.5 % of the amino acid content of collagen fibres; thus, the hydroxyproline content may reflect the formation of collagen in scar tissue [29]. Our data indicated that 0.2 mg/ml and 0.1 mg/ml RAPA could reduce intraarticular fibrous adhesion through inhibition of fibroblast collagen synthesis in a dose-dependent manner. In the animals treated with 0.2 mg/ml of RAPA, intraarticular adhesion became soft and weak around the decorticated areas of the femoral condyle macroscopically, which was consistent with the histological observation. The adhesion score, the hydroxyproline content and the fibroblast density were also significantly decreased compared with those of the control group. In the 0.1 mg/ml RAPA group, moderate adhesion tissues were found around the decorticated areas by macroscopical and histological observations. The hydroxyproline content and the fibroblast density were also less than those of the 0.05 mg/ml RAPA and control group. However, the 0.05 mg/ml RAPA group failed to reduce intraarticular fibrous adhesion through inhibiting fibroblast proliferation. Dense adhesions were obviously observed around the decorticated areas in the 0.05 mg/ml RAPA group. The adhesion score, the hydroxyproline content and the density of fibroblasts in 0.05 mg/ml RAPA group showed no significant differences compared with the control group. Therefore, these results showed that RAPA is an effective pharmaceutical agent for the prevention of intraarticular adhesion after knee surgery.

However, in the present study, we only investigated the effect of RAPA on reducing intraarticular adhesion by morphology and histology, which involved very basic scientific techniques and more in-depth studies should be conducted as a follow-up.

## Conclusions

The local application of RAPA could reduce intraarticular adhesion after knee surgery in rabbit models by inhibiting proliferation of fibroblasts and decreasing collagen synthesis, which may provide a new idea for reducing intraarticular adhesion after knee surgery in the clinical setting.

## Ethics approval

All of the animals received care according to the principles of Laboratory Animal Care of international recommendations and the experimental protocol was approved by the Animal Care and Research Committee of Central South University, China.

## Abbreviations

HE: haematoxylin and eosin
mTOR: a mammalian target of rapamycin
RAPA: rapamycin
